# The Need for an Evidence-Based Encyclopaedia in Health Services Research in Pharmacy

**DOI:** 10.3390/ijerph17072549

**Published:** 2020-04-08

**Authors:** Zaheer-Ud-Din Babar

**Affiliations:** Centre for Pharmaceutical Policy and Practice Research, Department of Pharmacy, University of Huddersfield, West Yorkshire HD1 3DH, UK; z.babar@hud.ac.uk

## Abstract

Pharmacy practice research (PPR) is a specialty field within the wider area of health services research and it focuses on studies of how and why people access pharmacy services. This stream of research is also referred to as more universally recognized term such as health services research in pharmacy. The health services research in pharmacy has increased manifold; however, the impact of this research is not visible at the global level. The editorial explains several issues on quality and quantity of evidence produced including how evidence produced could contribute to improve quality of care and patients’ health outcomes. It also narrates examples from the UK and Australia showing how health services research in pharmacy has made an impact on healthcare service delivery. The editorial argues that building an encyclopaedia in health services research in pharmacy is vital to enhance the visibility and impact of this research.

## 1. Introduction

As COVID-19 is threatening to change the course of healthcare systems, economies and countries, it has also emphasized the need for humanity to create credible scientific evidence to act upon in public policy space. A recent study [1] published by the Imperial College London changed the stance of the UK government towards COVID-19, changing it from “herd immunity” to a more “pragmatic approach”. In the field of pharmacy practice, the research has increased manifold; however, the impact of this research is less visible at the global level. This editorial discusses health services research in pharmacy highlighting how building an encyclopaedia or a major reference work (MRW) would be useful to enhance the visibility and impact of this research.

## 2. Pharmacy Practice Research or Health Services Research in Pharmacy

Pharmacy practice research (PPR) is a specialty field within the wider area of health services research and it focuses on studies of how and why people access pharmacy services, the cost of care and services and what happens to the patients as a result of this care [2]. Bond and Tsuyuki [3] have argued that pharmacy practice research components could also be referred to as more universally recognized terms such as applied health sciences or health services research in pharmacy.

In the recent past, the importance of “health services research in pharmacy” has increased manifold. Roberts and Kennington stated [4] that “Research is not just for academics but is about real issues that affect patients and pharmacists”. Many conditions that were previously managed in the hospital setting are now managed in primary care settings, and the research paradigm in this field is increasingly visible. 

The Royal Pharmaceutical Society of Great Britain’s publication, *New Medicines, Better Medicines, Better Use of Medicines* [5] has also emphasized the importance of “pharmacy practice research”. It stated that unless we do research, pharmacists’ central role will be compromised in the future. In 1997, Mays [6] stated that all pharmacists should be research aware, 10% should be research active and 1% should be research leaders. However, this strategy has not worked as clearly there are less than 500 pharmacy research leaders in both the United Kingdom and in Canada [3].

## 3. Evidence in Health Services Research in Pharmacy 

In health services research in pharmacy, the numbers of papers, publications and research articles are increasing. However, the quality is questionable, and the impact of this research is not visible at the global level. In the past, this research was criticized [7] because these were small descriptive, feasibility and proof of concept studies and they had little value in providing generalisable data. Moreover, the methodological approaches were not robust to generate the necessary evidence for policy change. The proliferation of small studies that did not generate new knowledge was not useful. More often many of these studies were surveys justifying just the role of the pharmacist [8].

In this context, the true nature and scale of this research and its impact on global health is not clear. Some of the pertinent questions are as follows: Has patient quality of life improved? Has this resulted in improved access to treatments or services? How and in what form and shape was the evidence used, and what policy decisions were based on research? 

## 4. Quantity and Quality of Evidence

There are a several issues that are related to the quality and quantity of evidence produced. For example, systematic reviews are largely used in evidence-based decision making, and they employ a rigorous approach to “reviewing” the literature in a well-defined way [9]. However, it is important to critically consider the quality of the review method [10] before reaching any conclusions. Although numbers of health services pharmacy reviews are increasing, very few are methodologically sound. For example, a literature search in the Cochrane library [11] shows that there are currently only 39 systematic reviews relevant to the pharmacy [12]. Moreover, in many of these reviews, the pharmacists are not the main focus. 

Cochrane reviews are excellent examples and they are increasingly used for evidence-based decision making. For example, a Cochrane review [13] on the effect of outpatient pharmacists’ non-dispensing roles on patient outcomes and prescribing patterns show a total of forty-three studies in this review. Thirty-six studies were pharmacist interventions targeting patients, while in seven studies pharmacist interventions were directed towards healthcare professionals. Only one study showed a significant improvement in systolic blood pressure for patients receiving medication management from a pharmacist compared to usual care from a physician. Pharmacists’ interventions resulted in improvement in most clinical outcomes, although these improvements were not always statistically significant. 

Another Cochrane review assessed the effectiveness of interventions by community pharmacists in smoking cessation [14]. There were few studies in this review and they suggested that the trained community pharmacists providing counselling may have had a positive effect on smoking cessation rates. However, the strength of the evidence was limited because only one of the trials showed a statistically significant effect. 

## 5. How is Evidence Contributing to Improve Care?

Above are a few examples depicting why methodologically sound studies are needed and what impact they could have on decision making. However, there is increasing evidence that pharmacists are delivering better care [8].

In Australia, the introduction of home medicine reviews (HMR) provides an interesting example of excellent integration between service provision and research [15]. An HMR involves a comprehensive medication review conducted by an accredited pharmacist. This research informed negotiations within the 3rd Community Pharmacy Agreement (CPA) to fund pharmacist and GP involvement in the Australia Home Medicines Review Programme. As a result, the sum allocated to fund professional pharmacy services under the five-yearly Community Pharmacy Agreements (CPA) has increased from $5 million in the 2nd CPA (1995–2000) to $663 million in the 5th CPA (2010–2015).

In the UK, a Cochrane review has provided information to build the government’s strategy to improve prescribing for older people in care homes [16]. In another example, a realist review was undertaken by using a realist, theory-driven approach to synthesize qualitative, quantitative and mixed-methods literature. This review contributes to a better understanding of how antimicrobial prescribing interventions for doctors-in-training can be embedded in the hierarchical and inter-professional dynamics of different healthcare settings [17].

The generation of evidence in health services research in pharmacy is considerably low from the low and middle-income countries (LMICs), both in quantity and quality [18]. The recent incidence of COVID-19 has very vividly brought to light the importance and need to strengthen the weak health systems. 

## 6. Building an Encyclopaedia

As researchers are performing pharmacy practice and health services research in all shapes and forms, it is perhaps time to document this in the form of an encyclopaedia. This would be a comprehensive major reference work documenting and narrating topics, areas, strategies, methodologies, research and theories in this field. The encyclopaedia would help us to see the gaps and to build a map of evidence and impact as well as a historical narrative on the topic. This would be a useful evidence-based resource to improve patients’ health outcomes.
